# Development of a novel surface assisted volume negative hydrogen ion source

**DOI:** 10.1038/s41598-017-10685-4

**Published:** 2017-09-11

**Authors:** B. Kakati, S. S. Kausik, M. Bandyopadhyay, B. K. Saikia, P. K. Kaw

**Affiliations:** 1 Centre of Plasma Physics-IPR, Nazirakhat, Sonapur-782 402, Kamrup(M), Assam India; 2 Institute for Plasma Research, HBNI, Bhat, Gandhinagar, 382 428 India

## Abstract

H^−^ ion based neutral beam injector is a critical heating and current drive system in a fusion reactor. However, the present H^−^ ion source configuration has limitations in terms of production, extraction, cesium (Cs) inventory and management. To overcome these limitations, a proof-of-principle experiment based on a novel concept regarding surface assisted volume H^−^ ions production by sprinkling Cs coated tungsten (W) dust grains (low work function surface) into a hydrogen plasma is carried out. Four different diagnostics have been used to validate the concept. The H^−^ ion fraction is estimated from (a) Langmuir probe diagnostic, (b) phase velocity of ion acoustic waves, (c) dust current and confirmed by the measurement of (d) Balmer line ratio. The measured H^−^ ion fraction with respect to the plasma density for different discharge conditions varies from ~0.2 to 0.3 in presence of Cs coated W dust particles. The experimental results show good agreement with the theoretical estimation.

## Introduction

Negative hydrogen ions (H^−^ ions) play an important role in many terrestrial^1–7^ as well as extra-terrestrial^8–10^ natural physical phenomena. The negative ions are often used in many other industrial applications, such as in ion beam deposition studies, for micrometer-sized powder surface modification, damage-free nano-particle formation for quantum devices, bio-compatibility surface treatment in nerve cell engineering^1^ fields, tandem accelerators^11^ and high energetic Neutral Beam Injection (NBI) system for fusion reactor^2–7^.

In last few decades, many researchers have paid tremendous efforts to develop a high current, low-emittance H^−^ ion sources to implement it in a NBI system^2–7^. NBI is one of the most reliable auxiliary heating and current drive system used in a fusion reactor for thermonuclear fusion^3, 4, 12, 13^. Thermonuclear fusion has the potential to produce sufficient energy to satisfy the mounting demand of energy of modern world. For thermonuclear fusion, it is necessary to heat the plasma above hundred million degrees centigrade. To heat the plasma, high energetic neutral atoms are injected into the plasma with the help of NBI system.

In a fusion grade NBI system, high current (~10 A) neutral beams having beam energy of ~1 MeV are required for plasma heating and current drive purpose. At that required energy of ~1 MeV, the neutralization efficiency for extracted H^−^ ions from the ion source is about 60%, whereas for H^+^ ion from a positive ion source, the neutralization efficiency tends to zero at energies above 200 kV. Thus, the fusion grade ion sources are based on negative ions instead of positive ions including in International Thermonuclear Experimental Reactor (ITER)-NBI design^2^. However, it is to be noted that the ITER grade negative ion source is still under developing stage through scaled up version^2–7^.

In laboratory, H^−^ ion can be produced either by binary collisional between plasma electrons and molecules based volume process or plasma ionic and atomic flux based surface process^12–15^. In the two step volume process, the H^−^ ions are produced through dissociative attachment (DA) of low energy electrons with the ro-vibrationally excited hydrogen molecules^4, 6^. On the other hand, in surface process, low work function surfaces are used for the production of H^−^ ions. When the impinging flux of hydrogen species (H and*/*or ${{\rm{H}}}_{x}^{+}$) from the hydrogen plasma collide with the low work function surface, they are reflected back as H^−^ ions by picking up electrons from the surface. The H^−^ ion production efficiency in surface process is significantly better than that of the collisional based two-step volume process^2–5^. However, volume process has some advantages due to its inherent isotropic nature.

At present, most of the high current H^−^ ion sources are operated in surface mode configuration, either cesium (Cs) vapour is injected into the plasma volume or Cs impregnated hot Cs dispenser^12–15^ is used to create low work-function surface^2^ inside the ion source. In surface process, Cs coated plasma grid (PG), made of tungsten (W) or molybdenum (Mo) is commonly used for H^−^ ion production which is placed close to the ion extraction system^2, 3^. The H^−^ ion conversion yield strongly depends on the work function of the PG which depends on the Cs coverage^16, 17^ on it. The surface conversion efficiency can be upto ~20% of the flux of incoming H atoms and ions^18, 19^.

However, despite the higher efficiency of surface process, there are some specific issues linked with uncontrolled behavior, which are: long-term operational stability, Cs pollution within accelerator stages and high Cs consumption that get buried under other sputtered wall material (e.g. Cu, W etc.)^16, 17, 20^. Another drawback for H^−^ ion extraction for the beam formation is linked with the initial direction of surface produced H^−^ ions on Cs coated PG surface. The vast bulk of the surface produced H^−^ ion start on trajectories that are directed towards the centre of the source (opposite to the extraction direction) due to the sheath electric field of the PG and gets electrostatically confined within the plasma. Schematic of the negative ion movement from the surface can be visualized in ref. 21. For the extraction, the direction of the H^−^ ions after the birth through surface conversion needs to be reversed which makes it difficult to extract them efficiently. Thus, to improve the extraction efficiency, the H^−^ ions should be produced uniformly in the plasma volume close to extraction aperture with higher production yield. This requirement leads to a concept of surface-assisted-volume negative ion source.

In this communication, we have explored the production of H^−^ ions, based on a novel technique by sprinkling micron sized Cs coated W dust through the surface production mechanism. The advantage of the present technique is that the probability of Cs pollution and high Cs consumption within accelerator stages is negligible. As the H^−^ ions are produced almost uniformly around the dust column in the plasma volume, thus the H^−^ ions are coming out from the dust surface isotopically and fill the plasma volume. As a result, it is not required to reverse the direction of H^−^ ions for the extraction. As the micron sized Cs coated W dust are used as low work function surface for H^−^ ion production, the present source has an advantage to improve the ratio of low work function surface area to plasma volume further by changing the dust density without changing the plasma setup geometry which will help to develop a compact efficient ion source in future. It needs to be emphasized that no such work (conceptually as well as experimentally) has been reported in the literature so far.

## Results and Discussions

Experimentally, the H^−^ ion fractions are measured using Langmuir probe and ion acoustic waves (IAW) in the present setup. A new approach to use dust as a diagnostic for the measurement of H^−^ ion fraction is also verified. For better confirmation about the production of H^−^ ions, the Balmer line ratio using optical emission spectroscopy (OES) is also studied.

To estimate the H^−^ ion density theoretically, a 0-D modelling on the basis of particle balance equation is carried out. Using the particle balance condition, the H^−^ ion density can be written as

1 $${n}_{-}=\frac{({n}_{d}{n}_{H}{A}_{d}{v}_{H}){\gamma }_{H}+({n}_{d}{n}_{{H}_{x}^{+}}{A}_{d}{v}_{{H}_{x}^{+}}){\gamma }_{{H}_{x}^{+}}}{{n}_{e}{\langle \sigma v\rangle }_{ED}+{n}_{{H}_{x}^{+}}{\langle \sigma v\rangle }_{MN}+{n}_{{H}_{2}}{\langle \sigma v\rangle }_{AD}+1/{\tau }_{-}}$$

Here, 〈*σv*〉_*ED*_, 〈*σv*〉_*MN*_ and 〈*σv*〉_*AD*_ are reaction rates for electronic detachments (ED), mutual neutralizations (MN) and associative detachments (AD). The parameters n_j_(j = −, e, +, d and H_2_) are the H^−^ ion density, electron density, ${{\rm{H}}}_{{\rm{x}}}^{+}$ ion density, dust density and H_2_ molecule density respectively. Similarly, the parameters *v*_j_ and γ_j_ (j = H, ${{\rm{H}}}_{{\rm{x}}}^{+}$) represent the thermal velocities and H^−^ ion conversion yields for H atoms and ${{\rm{H}}}_{{\rm{x}}}^{+}$ ions^18, 19^. A_d_ is the area of dust grain and τ_−_ is the H^−^ ions confinement time. The model is only to understand the physical mechanisms in the process within the plasma and to ensure that the observed results are indeed following the conceptual idea only and correlated with experimentally observed data for different operational parameters.

In the present work, the hydrogen plasma is produced by hot cathode filamentary discharge technique in a horizontal plasma chamber continuously (~40 minutes); whereas the W dust particles are coated *in-situ* in a vertical chamber placed above the plasma chamber. The details of the experimental set up are explained in the method section. The experiment is carried out at three operating pressures (2 × 10^−3^ mbar, 8 × 10^−4^ mbar and 4 × 10^−4^ mbar) for discharge currents 100–500 mA. In this report, the experimental findings for operating pressure 8 × 10^−4^ mbar are only reported which is found to be most optimum operating pressure in terms of maximum H^−^ ions density based on optimization of production and destruction mechanisms.

### H^−^ ion fraction measurement by Langmuir probe

**Figure 1:**
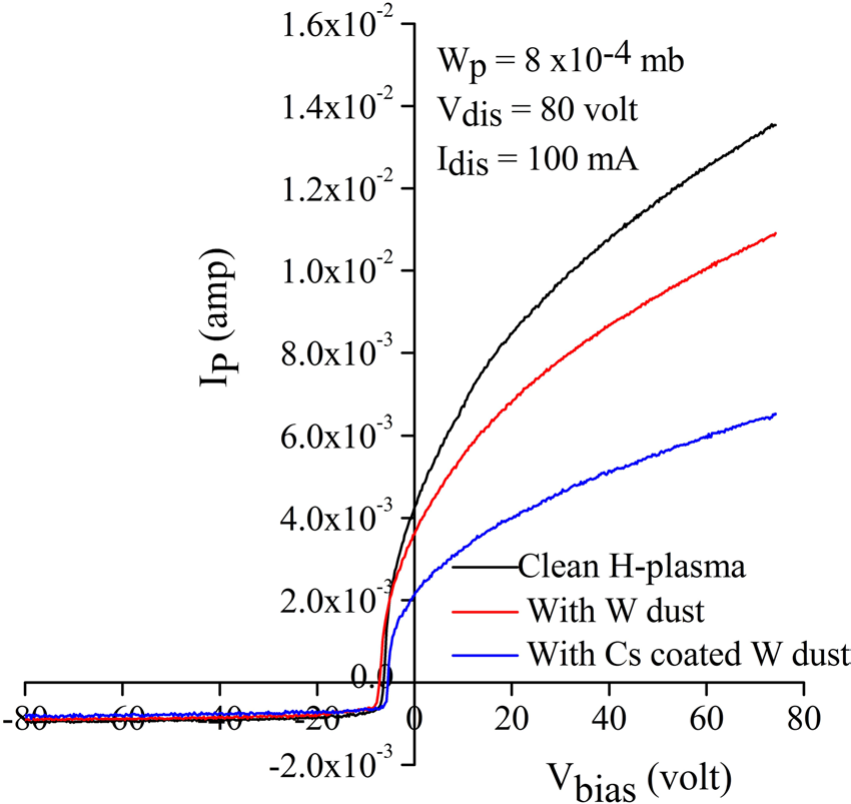
I-V characteristics of a Langmuir probe at different experimental conditions.

The typical I-V characteristics of a Langmuir probe for pristine hydrogen plasma, hydrogen plasma with uncoated and with Cs coated W dust are shown in Fig. 1.

**Figure 2:**
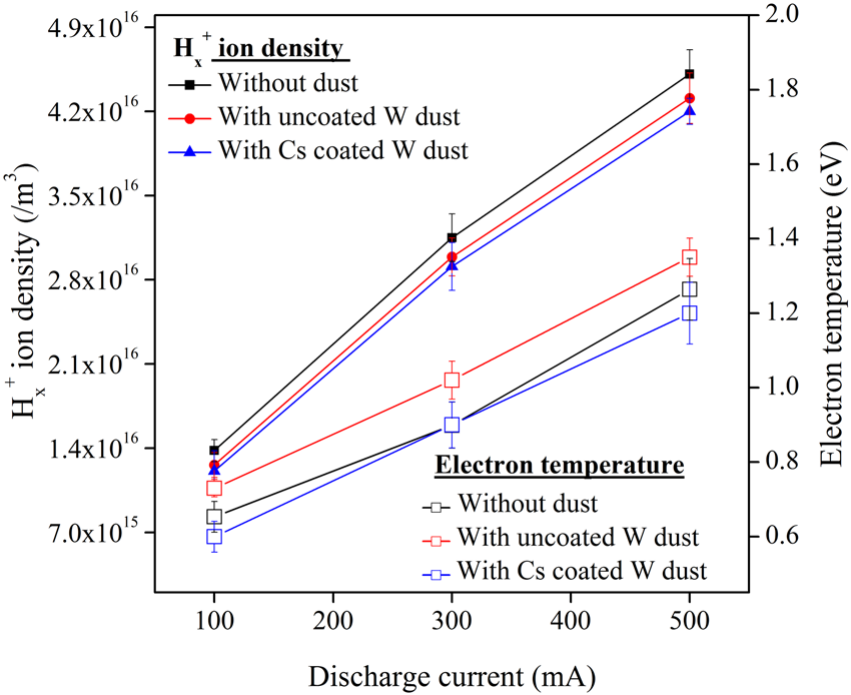
${{\rm{H}}}_{{\rm{x}}}^{+}$ ion density and electron temperature at different discharge conditions.

Plasma parameters are measured axially at 4.0 cm away from the centre of the magnetic cage such that dust grains do not touch the tip of the Langmuir probe during the dust operation. The ${{\rm{H}}}_{{\rm{x}}}^{+}$ ion densities and the corresponding electron temperatures for different discharge currents are shown in Fig. 2. Both the ${{\rm{H}}}_{{\rm{x}}}^{+}$ ion density and electron temperature increase with discharge current as expected due to higher ionization. The H^+^ ion density is calculated from the ion saturation current of the probe I-V characteristics. The electron temperature is derived from the transition region of the I-V characteristics. Both ${{\rm{H}}}_{{\rm{x}}}^{+}$ ion density and corresponding electron temperatures show almost identical behavior in all three cases. Variations in ${{\rm{H}}}_{{\rm{x}}}^{+}$ ion density and electron temperature in three cases are within the experimental error or uncertainty limit.

In Fig. 1, the probe current rising patterns tend to saturate for higher positive probe bias voltages. The probe current value at the plasma potential (V_p_) is considered as electron saturation current which can be determined by intersection method^22, 23^. It is seen that in presence of uncoated W dust, the electron saturation current decreases due to the loss of electrons on dust grains^24^. But in presence of Cs coated W dust, there is a further decrease of electron saturation current. The reduction of electron saturation current indicates the formation of H^−^ ions^25–27^ in presence of Cs coated W dust.

**Figure 3:**
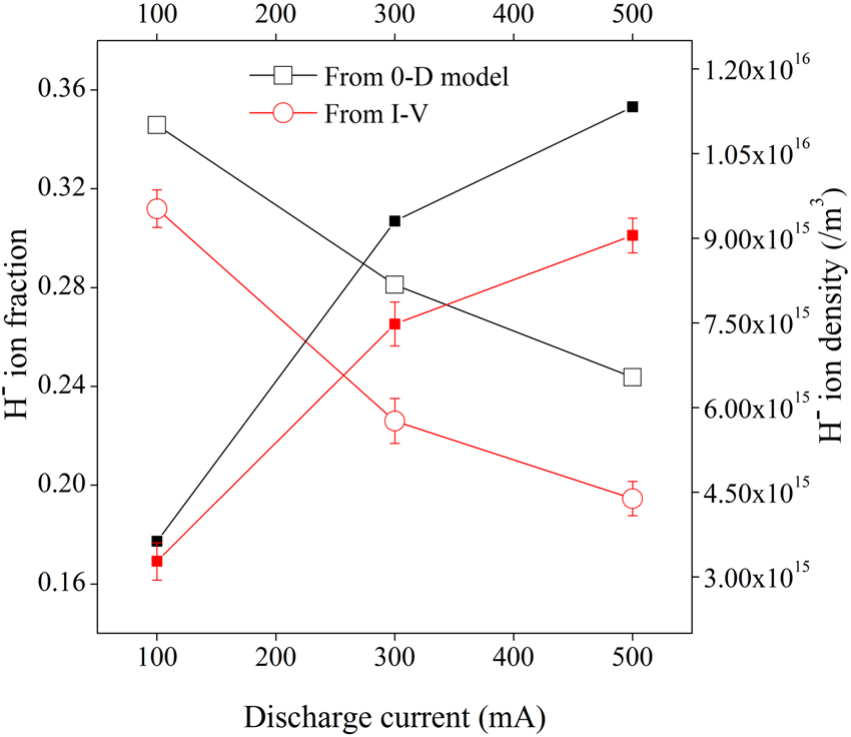
H^−^ ion fraction (open symbol) and H^−^ ion density (close symbol) at different discharge conditions.

Experimentally, the negative ion fraction (*ε*) is estimated from the reduction of the electron saturation current^25–27^ with the help of following equation^27^;

2 $$\varepsilon =\frac{{n}_{-}}{{n}_{+}}=1-\frac{{i}_{X2}^{Cs\_e}}{{i}_{X1}^{e}}$$

where ${i}_{X1}^{e}$ and ${i}_{X2}^{Cs{\rm{\_}}e}$ are the electron saturation current values in presence of uncoated and Cs coated W dust respectively. The H^−^ ion density and H^−^ ion fraction, measured by Langmuir probe using the Eq. 2 and calculated theoretically using Eq. 1 are shown in Fig. 3.

From the observations, it is seen that the Langmuir probe measurement and the theoretical estimation show a consistent trend. It is found that the H^−^ ion density increases linearly with the discharge current. When the discharge current increases from 100 mA to 500 mA, the plasma density increases from ~1.0 × 10^16^/m^3^ to ~4.5 × 10^16^/m^3^ and electron temperature increases from ~0.6 eV to ~1.2 eV. The increase in plasma density and temperature increases the flux of positive ions towards the negatively charged dust grains, which in turn increases the H^−^ ion density through surface process. But, the H^−^ ion fraction (ε) decreases with increase in discharge current. It is due to the increase of different destruction processes linked with the increase in plasma parameters as mentioned in our 0-D model.

Around ~10–15% lower H^−^ ion fraction (*ε*) is observed experimentally compared to the theoretical value, based on Eq. 1. The discrepancies can be explained from the aspect of few assumptions in the calculation. In our theoretical estimation, the H^−^ ion conversion yield (γ_j_) is considered as 0.1^18, 19^, based on close approximation of the energy of the ionic and atomic species of the plasma with the observations reported in refs 18 and 19. H atoms remain energetic ~1 eV for a considerable time after dissociation in a plasma volume due to its nature of elastic collisions with wall. In dusty plasma, the positive ions are accelerated within the sheath of a negatively charged dust grains. In the present experiment, the accelerated energy for positive hydrogen ions is found to be ~(4–5) eV (estimated using the dust surface potential profile). Therefore, H^−^ ion conversion yield for positive hydrogen ions and atoms as 0.1 is reasonable and conservative. However, it may be slightly different in real scenario, as experimental configurations are different. The degree of dissociation is considered as 10^−3^ in our theoretical estimation. However, these estimations may also be slightly different in actual experiment. The discrepancy between the theoretical and experimental values may also be within the measurement uncertainty of the plasma parameters.

### H^−^ ion fraction measurement from ion acoustics waves (IAW)

The IAW is used as an additional technique to estimate the H^−^ ion fraction in presence of Cs coated W dust grains in the present work. To measure the negative ion fraction using the phase velocity of IAW, a modified dispersion relation is derived for plasma with dust grains and H^−^ ions. Considering ${\omega }^{2}\langle \langle {v}_{te}^{2}{k}^{2},{\omega }^{2}\rangle \rangle {v}_{tj}^{2}{k}^{2}$ (j = +, − and d) and ${\omega }_{pd}\langle \langle \omega$ ^28, 29^, the dispersion relation for plasma with one positive ion species with density *n*_+_, one negative ion species with density *n*_−_ and negatively charged dust grains with density *n*_*d*_, can be written as,

3 $${(\frac{\omega }{k})}^{2}={C}_{s}^{2}\cdot \frac{1}{(1-\varepsilon -\delta {Z}_{d})}\cdot (1+\frac{\varepsilon }{M}){\rm{for}}\,{\rm{long}}\,{\rm{wave}}\,{\rm{length}}.$$

Here, ${c}_{s}=\sqrt{k{T}_{e}/{m}_{+}}$, $\delta ={n}_{d}/{n}_{+}$ and $M={m}_{-}/{m}_{+}$. Where T_e_ is the electron temperature, Z_d_ is the number of charge accumulated on dust grains and m_+(−)_ is the positive (negative) ion mass.

**Figure 4:**
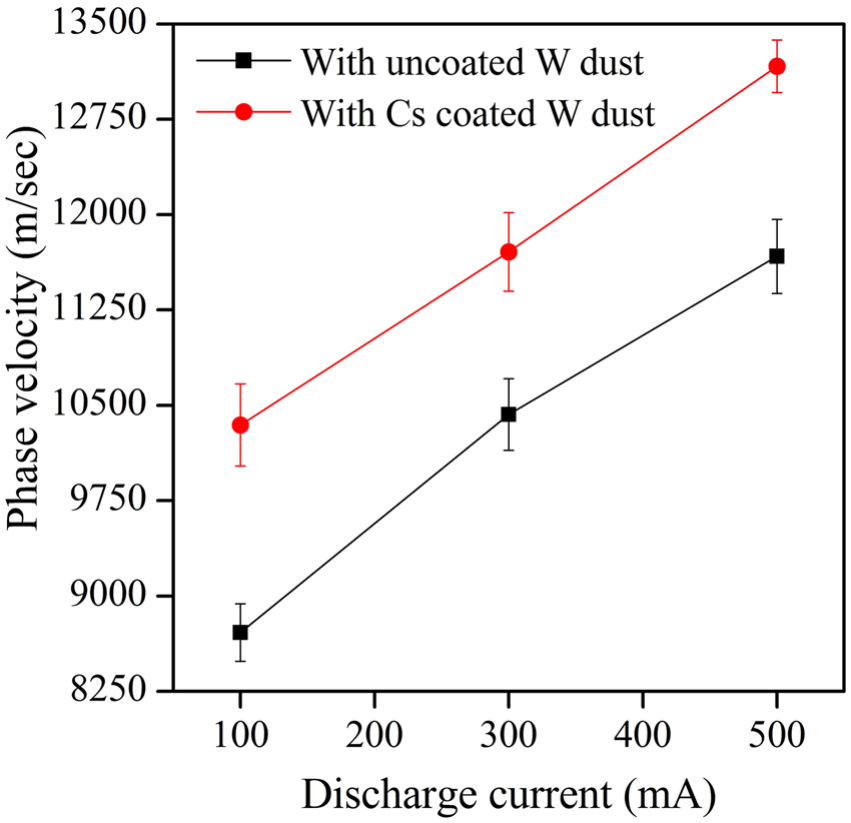
The phase velocity of IAW in presence of uncoated and Cs coated W dust.

In our present experiment, the phase velocity of IAW is measured using time of flight method, which is shown in Fig. 4. It is found that the phase velocity of IAW increases with discharge current for the cases of uncoated dust and Cs coated dust. The IAW phase velocity is more in presence of W dust grains compared to that of pristine plasma due to the reduction of electron density as a result of dust charging^29, 30^. The phase velocity further increases in presence of Cs coated W dust grains compared to uncoated W dust grains due to further reduction of electron population as a result of partial replacement of electrons by the presence of negative ions (in the negative space-charge portion in a quasi-neutral plasma). The enhancement of phase velocity confirms about H^−^ ion production in presence of Cs coated W dust grains.

**Figure 5:**
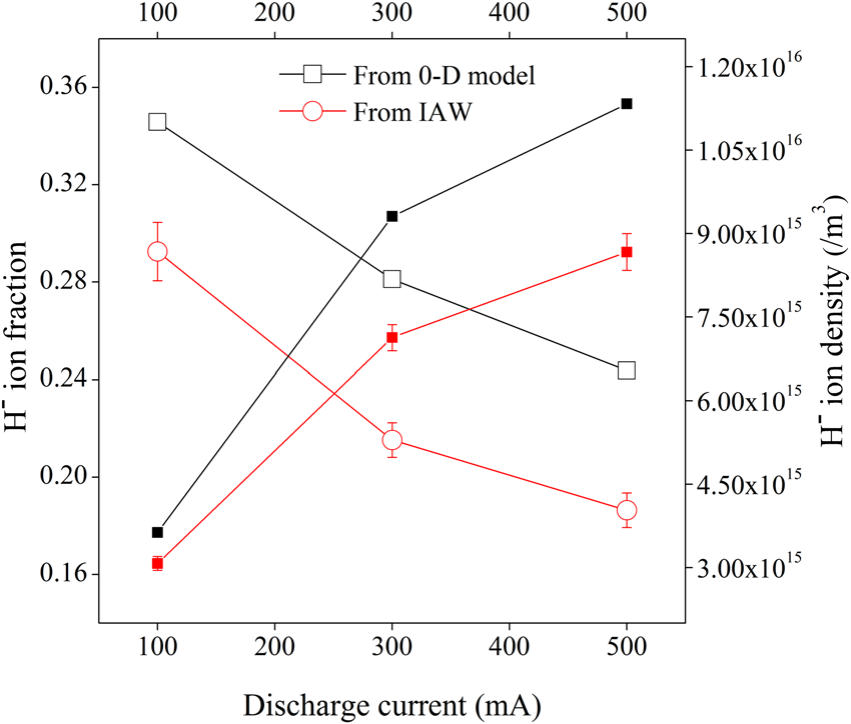
H^−^ ion fraction (open symbol) and H^−^ ion density (close symbol) at different discharge conditions.

The H^−^ ion fraction, measured from phase velocity of IAW using Eq. 3 at different conditions is shown in Fig. 5. From Fig. 5, it is seen that both Langmuir probe and IAW diagnostics results show a very good agreement about the production of H^−^ ion in presence of Cs coated W dust grains with the theoretical estimation (using Eq. 1).

### H^−^ ion fraction measurement from dust charge

The H^−^ ion fraction is measured by comparing the current carried by the dust particles with and without Cs coating. It is a new approach to use the charged dust grain as a diagnostic for measurement of negative ion fraction. In the present experiment, the current carried by the charged dust grains is measured with the combination of a Faraday cup and an electrometer which is explained in details in the method section of our manuscript. When the charged dust grain strikes the inner copper plate of a Faraday cup, a small current (in the range of nA) is registered by the electrometer. From the current, measured by the electrometer, the number of charges accumulated on dust grains (Z_d_) is estimated. The number of charges accumulated on dust grains is also calculated using capacitance model.

**Figure 6:**
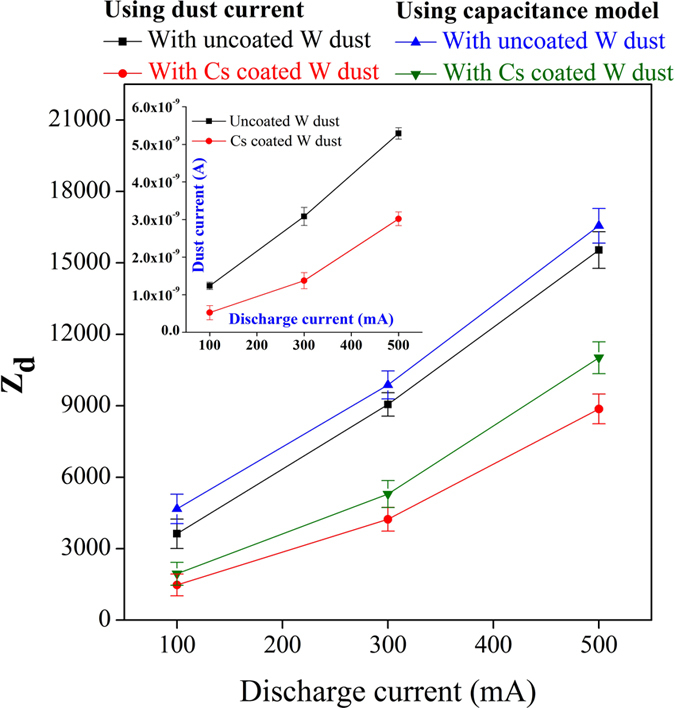
Dust charge and dust current (shown in inset) values for uncoated and Cs coated W dust.

Figure 6 shows the number of charges accumulated on uncoated and Cs coated W dust grains using capacitance model and dust current value. The dust currents for uncoated and Cs coated W dust are shown inset. It is seen that the number of charges accumulated on Cs coated W dust grains decreases significantly compared to the uncoated W dust grains. The decrease in dust charge in presence of Cs coated W dust grains reflects the production of H^−^ ion because of reduction in electron population in the plasma, as discussed before in Langmuir probe section.

The H^−^ ion fraction in presence of Cs coated W dust grain is estimated using the quasi-neutrality condition by substituting the value of Z_d_ with the help of the following relation;

4 $$\varepsilon =[1-(\frac{{i}_{X2}^{Cs{\rm{\_}}i}}{{i}_{X0}^{i}})(\frac{{i}_{X2}^{Cs{\rm{\_}}e}}{{i}_{X0}^{e}})]-\frac{{n}_{d}{Z}_{d}}{{n}_{+}}$$

where n_+_ is the ${{\rm{H}}}_{{\rm{x}}}^{+}$ ion density, n_d_ is the dust density, ${i}_{X0}^{i(e)}$ is the ion (electron) saturation current for pristine H^−^plasma, ${i}_{X2}^{Cs{\rm{\_}}i(e)}$ is the ion (electron) saturation current for H^−^plasma with Cs coated W dust grains and Z_d_ is the number of charges accumulated on dust grains. Detailed derivation is explained in the later part of the manuscript.

**Figure 7:**
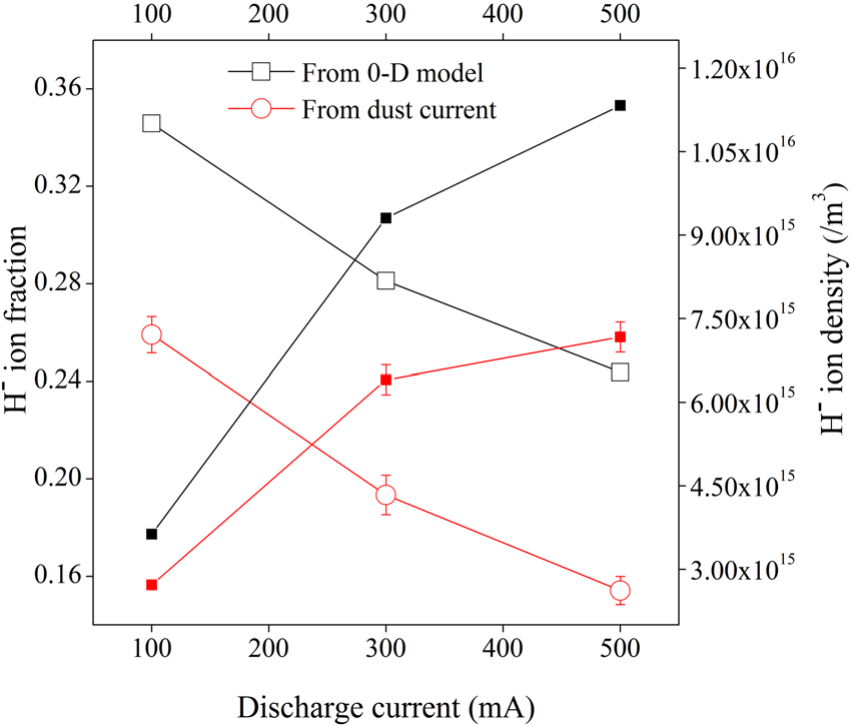
H^−^ ion fraction (open symbol) and H^−^ ion density (close symbol) at different discharge conditions.

The H^−^ ion fractions and the corresponding H^−^ ion density measured from dust current for different discharge currents are shown in Fig. 7. It is seen that the H^−^ ion fraction measured from dust current results also show good agreement with Langmuir probe and IAW results at different discharge currents.

### Measurement of H_α_/H_β_ line intensity ratio

**Figure 8:**
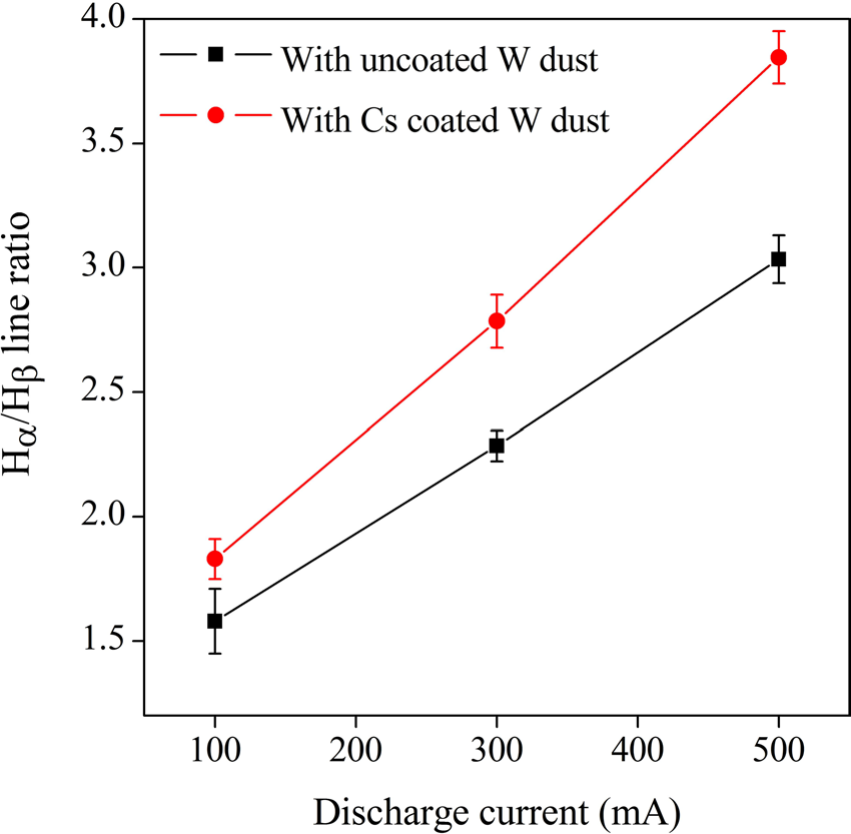
H_α_ and H_β_ spectral line intensity ratio.

Optical emission spectroscopy (OES) is used to monitor the Balmer lines and Cs lines in the present work. The H_α_/H_β_ ratio is used as an indicator to monitor the H^−^ ion production in presence of Cs coated W dust. The plasma is scanned for 350–870 nm to detect the intense Cs and H^−^Balmer lines continuously using a ½ m digikrom spectrometer (CVI Laser Corp, USA, Digikrom Model DK 480). The most intense neutral Cs spectral lines as well as the different ionic Cs lines are not observed during the entire experiment. It implies that partial concentration of Cs vapour in the plasma volume is insignificant. The line intensity ratio of H_α_/H_β_ is shown in Fig. 8.

It is seen that the line intensity ratio of H_α_/H_β_ increases in presence of Cs coated W dust than uncoated W dust. It is clearly visible from Fig. 1 that the electron density decreases (due to lower electron saturation current) in presence of Cs coated W dust grains. There is a slight decrease in electron temperature in presence of Cs coated W dust grains. Thus, mutual neutralization of H^−^ ions with positive ions is only the route for enhancement of H_α_/H_β_ line intensity ratio in our case^2^. Therefore, increase in H_α_/H_β_ line intensity ratio reveals about the production of H^−^ ion in presence of Cs coated W dust^2^. Since presence of Cs in the plasma volume is almost negligible, it can be implied that H^−^ ions are produced from Cs coated W dust grains only.

## Conclusion

In conclusion, the concept of a surface assisted volume negative ion source is realized in the recent experiment by sprinkling Cs coated W dust grains into the plasma. The production of H^−^ ions from Cs coated W dust is confirmed with the help of four independent diagnostics. The reduction of electron saturation current and increase in phase velocity in presence of Cs coated W dust grains reflect the production of H^−^ ions from Cs coated W dust grains. The increase in H_α_/H_β_ line ratio and decrease in dust charge for Cs coated W dust also indicate the production of H^−^ ion. OES measurement shows that there is no Cs vapour in the plasma volume which confirms that H^−^ ions are produced from Cs coated W dust grains only. From Langmuir probe, IAW and dust current measurements, it is found that the negative ion fraction is around 0.2–0.3 (20–30%) *w*.*r*.*t*. the plasma density (n_+_). The experimental results show a good agreement with the theoretical estimation. We would like to emphasize here that despite removing the difficulties of the conventional sources, the efficiency of the described novel negative ion source can be further enhanced by tuning several parameters, namely dust number density, cesium coverage on the dust grains, increase of plasma density, reduction of electron temperature etc. The present invention opens up a new and better direction to the scientific community to develop a new generation compact H^−^ ion source in near future.

The reported results obtained in a small prototype experimental device confirm that the present technique for H^−^ ion production has potential to be used to make a high current negative ion source. However, several technical and interface issues, *e.g.* beam extraction possibility, negative ion production over large area which is linked with the beam uniformity, dust consumption and its management etc. need to be worked out. To address these issues, a long term prototype experimental plan is being carried out. The next phase of the experiment is the extraction of H^−^ ions as a beam from the present prototype source to validate the ion source concept. In parallel, for accurate localized H^−^ ion measurement, a laser photo-detachment diagnostic is also under process to address negative ion density profile around the dust column. After the extraction phase, uniformity of H^−^ ion production over a large area will be attempted using multiple Cs coated W dust columns. The conceptual design of each experimental phase is being carried out and corresponding experimental results will be reported in near future.

## Methods

**Figure 9:**
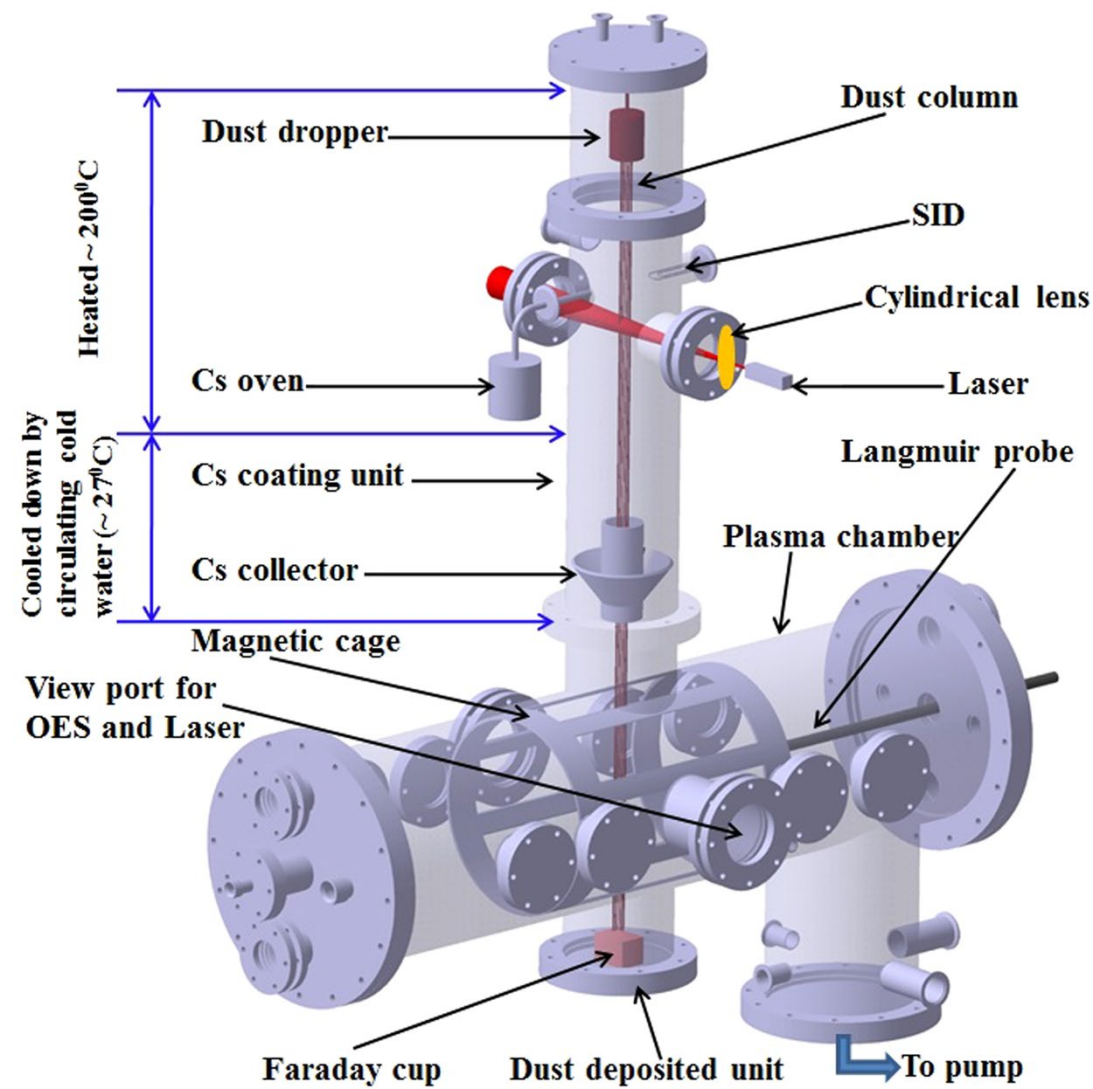
The 3-D view of the experimental set up.

A 3-D view of the present experimental device, used for the production of H^−^ ions is shown in Fig. 9. It consists of two stainless steel chambers placed one above the other in orthogonal configuration. The horizontal chamber is the plasma chamber (Length: 100 cm and Dia: 30 cm) and vertical upper chamber is the Cs coating unit (Length: 72 cm and Dia: 15 cm). The Cs coating unit (CCU) to coat the W dust *in-situ* by Cs comprises of a W dust dropper, Cs oven and different diagnostics ports with heating and cooling facilities. Cs vapour density in CCU is monitored by a surface ionization detector (SID)^31^. The description of SID can be obtained in ref. 31.

The CCU is designed in such a way that only the Cs coated W dust particles can enter and pass through the horizontal chamber, *i*.*e*. plasma chamber. The upper portion of CCU’s wall is heated up to 200 °C for better recycling the Cs vapour. Cold water (~12 °C) is circulated through the lower portion of CCU’s wall to condense the Cs vapour. Additionally, a Cs collector is used at the bottom of the CCU to trap the Cs vapour such that it cannot enter into the plasma chamber directly. The condensed Cs is deposited on the Cs collector.

The W dust grains having average sized ~ 3.0 µm with average weight 10^−13^ kg are dropped from the top of the CCU by agitating a mess sieve with the help of a DC motor. The W dust grains are allowed to pass through a Cs vapour column before entering into the plasma chamber through the opening of the Cs collector (Fig. 9). As a result, the W dust grains are coated with Cs. The Cs coverage on W dust grain is estimated theoretically considering the transit time (~ms) of dust grain in the Cs column, Cs flux and total numbers of Cs atom to form a Cs monolayer on W dust. From theoretical estimation, it is found that the Cs coverage on W dust grain is ~0.4 monolayer which corresponds to the work function for Cs coated W dust particle is ~1.8 eV^32^. The dust-dropping rate is controlled by the vibration frequency of the DC motor, which is mechanically coupled with the dust dropper. The dust density is measured continuously using the laser scattering technique^33^ which is found to be of the order of 10^11^/m^3^ in the present work. The dust density is measured in two different locations of the experimental set up. To measure dust number density at CCU, a solid state semiconductor laser (Power: 5 mW, wavelength: 650 nm and dimension of laser beam: Approx. 8 mm at 5 meter) is mounted on a stand in front of the view port of the CCU. Whereas the dust density inside the plasma volume is measured by mounting the laser in front of the central view port of the plasma chamber which is mostly used for OES. It is to be noted that since tungsten dusts are heavy, gravitational force acting on it is significant and fall freely through the plasma without getting trapped in the plasma by electrostatic forces.

The experimental set up is evacuated up to a base pressure of 2 × 10^−6^ mbar with the help of a diffusion pump backed by a rotary pump. Ultra pure hydrogen gas is fed into the plasma chamber with the help of a digital flow controllers (DFC) (*AALBORG* make) attached to the plasma chamber. Hydrogen plasma is produced by striking a discharge between incandescent tungsten filaments and the grounded magnetic cage, which serves as anode. In the present work, two incandescent thoriated tungsten filaments of total length 150 mm and diameter 0.25 mm are used. The filament heating current is ~6A each (max). Ionizing electrons emitted from hot tungsten filaments are accelerated by applying 80 volt discharge voltage^34^. A multi-dipole magnetic cage consisting of cubical strontium ferrite magnets of surface field ~1.2 kG, which are arranged in a cylindrical shape, is used for plasma confinement.

Usually, the measurement of negative ion density has been performed experimentally utilizing different techniques like a Langmuir probe^25–27^, the propagation of ion acoustics waves (IAWs)^28, 35^ and optical emission spectroscopy (OES)^2^. Additionally, the H^−^ ion fraction is also estimated using the dust current in the present work. It is to be noted that all the above diagnostics which are employed to measure the H^−^ ion density can give only the orders of magnitude and not possible for accurate measurements of H^−^ ion density. The prime objective of the present experiment is to validate the novel route of H^−^ ion production from Cs coated W dust particles. The measurements indeed prove the concepts and show the influence of different operational parameters on negative ion production. However, for accurate measurement of H^−^ ion density a laser photo-detachment diagnostic setup is under process to be installed in the system.

It is well known that the H^−^ ion production in surface process directly depends on the low work function surface area. The low work function surface area in the present plasma experiment can be increased by increasing the dust density, size of the dust grains and diameter of the dust column. Considering the simple geometry of the dust column (dust radius, dust column radius and height), plasma chamber (radius and height) along with the dusty plasma characteristic parameters (dust potential, dust charge and Debye length), the relative surface area can be expressed as,

5 $$\frac{{A}_{D}}{{A}_{ch}}=\frac{{r}_{d}}{{R}_{ch}}\frac{{h}_{dc}}{{h}_{ch}}{(\frac{{r}_{dc}}{{\lambda }_{D}})}^{2}$$

Assuming $\frac{{Z}_{d}{e}^{2}}{{r}_{d}}=3{T}_{e}$ and ${n}_{d}{Z}_{d}\approx {n}_{e}$ [Considering the case for maximum number of dust particles in plasma volume as a limiting case] for dust containing hydrogen plasma]. Where A_D_, A_ch_, r_d_, R_ch_, h_dc_, h_ch_, r_dc_, λ_D_ are the area of dust column, area of plasma chamber, radius of dust grains, radius of plasma chamber, height of dust column, height (diameter) of the plasma chamber, radius of dust column and Debye length respectively. The Eq. 5 indicates that the dust surface area becomes significantly larger than the cross sectional area of the plasma chamber. Moreover, few optimization knobs are available to further increase the dust surface area. The higher value of relative surface area confirms that the present idea may pave the way to develop a compact efficient negative ion source in future.

### Theoretical model for estimation of H^−^ ion fraction

To estimate the H^−^ ion density theoretically and to understand the experimental results, a theoretical model based on particle balance condition is developed, considering different H^−^ ion production and destruction routes. The model is not applicable for independent calculation of H^−^ ion fraction. The model is only to understand the physical mechanisms in the process within the plasma and to ensure that the experimentally observed results are indeed following the conceptual idea for different operational parameters. In the present experiment, the H^−^ ion production yield through volume process is considerably low^22, 36–38^ and thus it is neglected in the theoretical estimations. In volume H^−^ ion source, approximately 90% of the negative ions are generated from the ${H}_{2}^{\ast }(\nu ^{\prime\prime} )$ (14 ≥ *ν*″ ≥ 5) molecules through high energy electron vibrational excitation **(**E-V) reaction process. This process is efficient only if high energy electrons (>10 eV) collide with ground electronic state of hydrogen molecules X^1^Σ_g_^+ ^^39–41^. For efficient H^−^ ion production through volume process, two separate plasma regions, populated with high and low energetic electrons are required. There is no such separate region, populated with high and low energetic electrons in our present experimental device. In the present experiment, it is observed that the electron temperature is very low (~1 eV) for the entire experimental conditions and the electron energy probability function (EEPF) shows that the high energy electron population (with energy >10 eV) is remarkably low. As per present experimental condition, the primary electron fraction is ~10^−3^ w. r. to the bulk plasma density. The primary electron fraction is consistent with the assumed dissociation and ionization fraction in the model. Thus, the formation of H^−^ ion through volume process is considered as insignificant compared to surface production process in our present work.

As the Cs coated W dust grains are used for the production of H^−^ ions, only the surface process is considered as production route in our case. The electronic detachments (ED), mutual neutralizations (MN) and associative detachments (AD) are the main destruction route of H^−^ ions^22, 36–38^ and are incorporated in our model.

Thus, the 0-D particle balance rate equation for H^−^ ion production can be written as;

6 $$\begin{array}{c}{n}_{-}{n}_{e}{\langle \sigma v\rangle }_{ED}+{n}_{-}{n}_{{H}_{x}^{+}}{\langle \sigma v\rangle }_{MN}+{n}_{-}{n}_{{H}_{2}}{\langle \sigma v\rangle }_{AD}+\frac{{n}_{-}}{{\tau }_{-}}=({n}_{d}{n}_{H}{A}_{d}{v}_{H}){\gamma }_{H}+({n}_{d}{n}_{{H}_{x}^{+}}{A}_{d}{v}_{{H}_{x}^{+}}){\gamma }_{{H}_{x}^{+}}\\ {n}_{-}({n}_{e}{\langle \sigma v\rangle }_{ED}+{n}_{{H}_{x}^{+}}{\langle \sigma v\rangle }_{MN}+{n}_{{H}_{2}}{\langle \sigma v\rangle }_{AD}+1/{\tau }_{-})=({n}_{d}{n}_{H}{A}_{d}{v}_{H}){\gamma }_{H}+({n}_{d}{n}_{{H}_{x}^{+}}{A}_{d}{v}_{{H}_{x}^{+}}){\gamma }_{{H}_{x}^{+}}\end{array}$$

Here, left hand side (LHS) represents total destruction rate and the right hand side (RHS) corresponds to the total surface production rate. The terms 〈σv〉_ED_, 〈σv〉_MN_ and 〈σv〉_AD_ are the reaction rates for ED, MN and AD reactions. The parameters n_j_(j = −, e, +, d and H_2_) are the H^−^ ion density, electron density, ${{\rm{H}}}_{{\rm{x}}}^{+}$ ion density, dust density and H_2_ molecule density respectively. Similarly, the parameters *v*_j_ and γ_j_ (j = H, ${{\rm{H}}}_{{\rm{x}}}^{+}$) represent the thermal velocities and H^−^ ion conversion yields for H atoms and ${{\rm{H}}}_{{\rm{x}}}^{+}$ ions^18, 19^ which are almost same and is considered as 0.1 respectively. The first term of the RHS of particle balance rate equation (Eq. 6) represents the atomic contribution and second term represents the ionic contribution for H^−^ ion production from Cs coated W dust. As the degree of dissociation in low pressure hydrogen plasma is very low^42, 43^, the atomic density is considered as 10^−3^ times of hydrogen molecular density (${n}_{H}\approx {10}^{-3}{n}_{{H}_{2}}$) in the present calculation. The τ_−_ is the H^−^ ions confinement time^37, 44^. The reaction rates for ED, MN and AD reactions are estimated theoretically for our case following methods reported by many authors^38, 45, 46^.

### H^−^ ion fraction measurement by Langmuir probe

Experimentally, the negative ion fraction (*ε*) is estimated from the reduction of the electron saturation current^25–27^ which are measured by a Hiden Analytical Limited’s ESPION Langmuir probe system in the present work. The probe tip is single and cylindrical having a diameter 0.15 mm and length 10.0 mm. The *ε* can be estimated using Langmuir probe with the help of the following equation^27^;

7 $$\varepsilon =\frac{{n}_{-}}{{n}_{+}}=1-\frac{{i}_{X1}^{i}}{{i}_{X2}^{Cs\_i}}\frac{{i}_{X2}^{Cs\_e}}{{i}_{X1}^{e}}\sqrt{\frac{{m}_{+(X1)}}{{m}_{+(X2)}}}{\rm{\Omega }}$$

where ${i}_{X1}^{i(e)}$ is the ion (electron) saturation current in presence of uncoated W dust and ${i}_{X2}^{Cs\_i(e)}$ is the ion (electron) saturation current in presence of Cs coated W dust. The term Ω is the sheath factor and $\frac{{m}_{+(X1)}}{{m}_{+(X2)}}$ is the positive ion mass ratio (which is considered as ~1) in presence (for X2) and absence (for X1) of H^−^ ions.

A large number of experiments and numerical simulations have been performed by different researchers on the sheath structures in electronegative gas plasma^46–48^. Shindo *et al*.^27^ reported that the sheath factor (Ω) can be evaluated from ${\rm{\Omega }}=(1-{\varepsilon }_{IAW})/(1-{\varepsilon }_{\Pr obe})$, where ${\varepsilon }_{X}$ represents the negative ion fraction measured by IAW and Langmuir probe technique. In the present experiment, the sheath factor (Ω) and ion saturation current ratio $\frac{{i}_{X1}^{i}}{{i}_{X2}^{Cs\_i}}$ is close to 1 based on the measurement. Thus, the Eq. 7 can be written in the form of Eq. 2.

### H^−^ ion fraction measurement from ion acoustics waves (IAW)

In the present work, IAW is used as an additional technique to estimate the H^−^ ion fraction^28, 35^. Generally, in presence of dust grains^29, 30^ and negative ion^28, 49^, the wave propagation significantly gets modified from the normal two-component plasma. The IAW splits into two modes in presence of negative ions: a fast and a slow mode. Cooney *et al*.^49^ reported that the speed of one branch (fast mode) increases and the speed of the other branch (slow mode) decreases with the increase of negative ion fraction *ε*. The fast mode is very sensitive to *ε*. In cold plasma, the slow mode does not exist. Thus, only the fast mode of IAW is observed in our recent experiment.

To estimate the negative ion fraction using the phase velocity of IAW, a modified dispersion relation is derived for plasma with dust grains and H^−^ ions on the basis of continuity equation, the momentum equation and Poisson’s equation^28, 50^. For plasma with one positive ion species having density *n*_+_, one negative ion species with density *n*_−_ and negatively charged dust grains with density n_d_, the dispersion relation can be written as,

8 $$1=\frac{{\omega }_{Pe}^{2}}{{\omega }^{2}-{v}_{te}^{2}{k}^{2}}+\frac{{\omega }_{P+}^{2}}{{\omega }^{2}-{v}_{t+}^{2}{k}^{2}}+\frac{{\omega }_{P-}^{2}}{{\omega }^{2}-{v}_{t-}^{2}{k}^{2}}+\frac{{\omega }_{Pd}^{2}}{{\omega }^{2}-{v}_{td}^{2}{k}^{2}}$$

where *ω*_*Pj*_ is the plasma frequency and *v*_*tj*_ is the thermal velocity for the respective species of j (j = electron, positive ion, negative ion and dust grains).

Considering ${\omega }^{2}\langle \langle {v}_{te}^{2}{k}^{2},{\omega }^{2}\rangle \rangle {v}_{tj}^{2}{k}^{2}$ (j = +, − and d) and ${\omega }_{pd}\langle \langle \omega$ ^28, 29^, the dispersion relation can be written in the form of Eq. 3.

The ion-acoustic perturbation is excited by applying a tone burst signal using a stainless steel (SS) mesh (50 lines per inch) with the help of a function generator through a dc blocking capacitor. The density perturbation as a fluctuation in the ion saturation current is detected using a co-axially placed movable planer Langmuir probe (dia: 3 mm) which is biased negatively. The phase velocities of the IAW are measured using time of flight method for pristine hydrogen plasma, hydrogen plasma with uncoated and Cs coated dust grains. The phase velocities are obtained by a linear fit to the distance vs time plot.

### Measurement of dust charge and H^−^ ion fraction

In laboratory, the dust grains are generally negatively charged mainly due to collection of electrons from the background plasma^24, 29, 51, 52^. Many researchers observed that the charge accumulated on dust grains decreases in presence of negative ions^53–55^.

The dust charge can be calculated theoretically with the help of capacitance model^24, 56^. Using capacitance model, the dust charge can be calculated using the relation given below:

9 $${q}_{d}=e{Z}_{d}=4\pi {\varepsilon }_{0}{r}_{d}{\varphi }_{d}$$

where “r_d_” is the radius of the spherical dust grain and ϕ_d_ is the dust surface potential which is estimated from the floating potential and plasma potential values, measured by Langmuir probe. By measuring the dust surface potential, the number of charges accumulated on dust grains can be estimated using the above relation.

**Figure 10:**
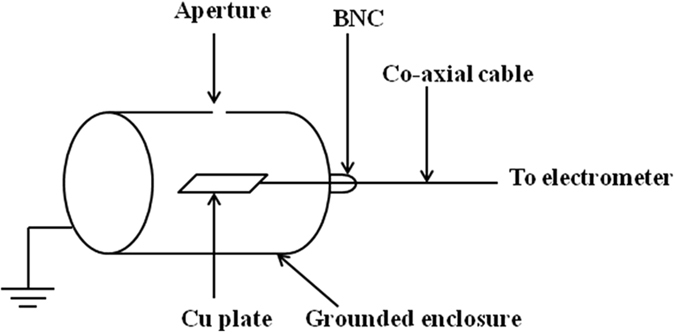
Schematic of Faraday cup.

In the present work, the number of charges accumulated on dust grains are estimated from the capacitance model and dust current measured experimentally) profile. A sensitive electrometer (Keithley Instruments, 6514), attached to the Faraday cup (FC) is used to measure the dust charge carried by a single dust in terms of current^34, 51, 57^. The FC assembly mainly comprises of a floating copper plate, which is encircled by a grounded cylindrical stainless steel shield. The Faraday cup is connected with the electrometer by using a low noise tri-axial cable. The schematic of FC is shown in Fig. 10. The entrance pinhole of the FC is 2 mm in diameter.

The FC is placed below the magnetic cage as shown in Fig. 9. The outer shell of the FC is electrically grounded. Apart from that, a strong transverse magnetic field is created by two permanent magnets of surface field strength 1.2 k G near FC aperture to prevent any lighter charged particle like electrons and ions to enter into the FC. The Larmor radius of micron sized dust particle (mass ~10^−13^ kg) is very large and deflection of it by magnetic field is very small and can be neglected. The incoming charged dust strikes the copper plate inside the FC and the charge flows to the ground through electrometer producing the dust current (~nA), which can be directly measured from the electrometer reading. The electrometer can be used to measure up to 1200 readings per second with fast integration or 17 measurements per second with 60 Hz line-cycle integration (as per model specifications) whose resolution is of the order of 10^−15^ A.

The current produced in the electrometer due to the charged dust grains, *i.e.* the dust current can be written as

10 $${I}_{d}={n}_{d}{Z}_{d}eA{V}_{d}$$

where “e” is the elementary charge, A is the charge collecting area and V_d_ is the drift velocity. Using the above relation, the number of charge accumulated on dust grain is estimated in the present work.

The H^−^ ion fraction in presence of Cs coated W dust a grain is estimated using the quasi-neutrality condition by substituting the value of Z_d_. For dust containing plasma without negative ions, the quasineutrality condition^54, 55^ can be written as

11 $${n}_{i}={n}_{e}+{n}_{d}{Z}_{d}$$

But, in presence of negative ions, the quasineutrality condition becomes

12 $$\begin{array}{ccc}{n}_{i} & = & {n}_{e}+{n}_{-}+{n}_{d}{Z}_{d}\\  & \Rightarrow  & {n}_{d}{Z}_{d}={n}_{i}(1-\frac{{n}_{e}}{{n}_{i}}-\frac{{n}_{-}}{{n}_{i}})\\  & \Rightarrow  & {n}_{i}\varepsilon ={n}_{i}[1-(\frac{{i}_{X2}^{Cs{\rm{\_}}i}}{{i}_{X0}^{i}})(\frac{{i}_{X2}^{Cs{\rm{\_}}e}}{{i}_{X0}^{e}})]-{n}_{d}{Z}_{d}\end{array}$$

where ${i}_{X0}^{i(e)}$ is the ion (electron) saturation current for pristine H^−^plasma, ${i}_{X2}^{Cs{\rm{\_}}i(e)}$ is the ion (electron) saturation current for H^−^ plasma with Cs coated W dust grains, Z_d_ is the number of charges accumulated on dust grains and $\varepsilon$ is the H^−^ ion fraction.

### Measurement of H_α_/H_β_ line intensity ratio

For *in-situ* non-invasive monitoring of H^−^ ion production yield, the H^−^ Balmer lines and Cs lines are monitored continuously. Considering the important excitation channels for H^−^ Balmer line emission^2^, the excited atomic hydrogen, *n*_*H*_(*p*) is

13 $${n}_{H}(p)={n}_{H}{n}_{e}{R}_{H}(p)+{n}_{{H}^{+}}{n}_{e}{R}_{{H}^{+}}(p)+{n}_{{H}_{2}}{n}_{e}{R}_{{H}_{2}}(p)+{n}_{{H}_{2}^{+}}{n}_{e}{R}_{{H}_{2}^{+}}(p)+{n}_{{H}^{-}}{n}_{{H}^{+}}{R}_{{H}^{-}}(p)$$

where *R*_*s*_(*p*) (*s* denotes the particle species) is the population coefficients.

Fantz and Wünderlich^2^ reported that the mutual neutralization process populates selectively the quantum number *p* = 3 with an enhancement of one order of magnitude in comparison to the quantum numbers *p* = 2 and *p* = 4. As a result, the H_*α*_ line intensity increases prominently in presence of H^−^ ions due to mutual neutralization reaction process between negative ions and positive ions. Thus, the line intensity ratio of H_α_/H_β_ is very sensitive to H^−^ ion density which is increased with the increase of H^−^ ion density.
